# *Eye and Vision* reviewer acknowledgement 2015

**DOI:** 10.1186/s40662-016-0039-5

**Published:** 2016-03-16

**Authors:** Jia Qu

**Affiliations:** Wenzhou Medical University, Wenzhou, China

## Abstract

The editors of *Eye and Vision* would like to thank all our reviewers who have contributed to the journal in volume 2 (2015).

**Omid Abouali**

Iran

**Renato Ambrósio Jr**

Brazil

**Veronique Andrieu**

France

**Steve Arshinoff**

Canada

**Magdalena Asejczyk**-**Widlicka**

Poland

**George Asimellis**

Greece

**Shady Awwad**

Lebanon

**Marcelo Ayala**

Sweden

**Ali Ayata**

Turkey

**Ewa Bartnik**

Poland

**Marcus Blum**

Germany

**Edmondo Borasio**

Italy

**Luca Buzzonetti**

Italy

**Gianluca Carifi**

United Kingdom

**Kenneth Cohen**

United States of America

**Marisol Corral**-**Debrinski**

France

**Baptiste coudrillier**

United States of America

**Vasilios Diakonis**

United States of America

**Mohamed Tarek Elnaggar**

Egypt

**Juan Fernandez de Castro**

United States of America

**Mariann Fodor**

Hungary

**Marianna Foldvari**

Canada

**Emre Güler**

Turkey

**Asaad Ghanem**

Egypt

**David Goldblum**

Switzerland

**Hovhannes Gukasyan**

United States of America

**Rasmus Søgaard Hansen**

Denmark

**Andreas Hartwig**

Germany

**Hesam Hashemian**

Islamic Republic Of Iran

**Kathryn Hatch**

United States of America

**Lawrence Hirst**

Australia

**Zibing Jin**

China

**Björn Johansson**

Sweden

**Maarten Kamermans**

Netherlands

**Savleen Kaur**

India

**Nathan Kerr**

Australia

**Karthikeyan Kesavan**

India

**Laura Kimpton**

United Kingdom

**Maria Kugelberg**

Sweden

**Jacky Lee**

Hong Kong

**Eitan Livny**

Israel

**Rizwan Malik**

United Kingdom

**Aifric Martin**

Australia

**Antonio Martínez**

Spain

**Cosimo Mazzotta**

Italy

**Jodhbir Mehta**

Singapore

**Ashim K Mitra**

United States of America

**Majid Moshirfar**

United States of America

**Mitsuru Nakazawa**

Japan

**Gabor Nemeth**

Hungary

**Kelechi Ogbuehi**

Saudi Arabia

**Christoffer Ostri**

Denmark

**Marco Piccirelli**

Switzerland

**Luisa Pierro**

Italy

**Paul**-**Rolf Preußner**

Germany

**António Queirós**

Portugal

**Norlina Ramli**

Malaysia

**Johann M.G. Reyes**

Philippines

**Tommaso Rossi**

Italy

**Ali Osman Saatci**

Turkey

**Gerald Schmidinger**

Austria

**Alexander Schuster**

Germany

**Vijay Setaluri**

United States of America

**Mohammad Shahidullah**

United States of America

**Ian Sigal**

United States of America

**Trefford Simpson**

Canada

**Rishi Singh**

United States of America

**Michael Stewart**

United States of America

**Nayyirih Tahzib**

Netherlands

**Lawrence Tam**

Ireland

**James Tsai**

United States of America

**Mayumi Ueta**

Japan

**Canan Asli Utine**

Turkey

**Ali Vahdati**

United States of America

**Keith Walter**

United States of America

**Xiaofei Wang**

Australia

**Keryn Williams**

Australia

**David Zadok**

Israel

